# Supplementary material for: Retinol dehydrogenase 10 reduction mediated retinol metabolism disorder promotes diabetic cardiomyopathy in male mice
Source: Nat Commun. 2023 Mar 2;14:1181. doi: 10.1038/s41467-023-36837-x (PMC9981688; doi:10.1038/s41467-023-36837-x)
Supplement: Supplementary file 1 — Supplementary Information [file 41467_2023_36837_MOESM1_ESM.pdf]

Supplementary Information

Supplementary Figures

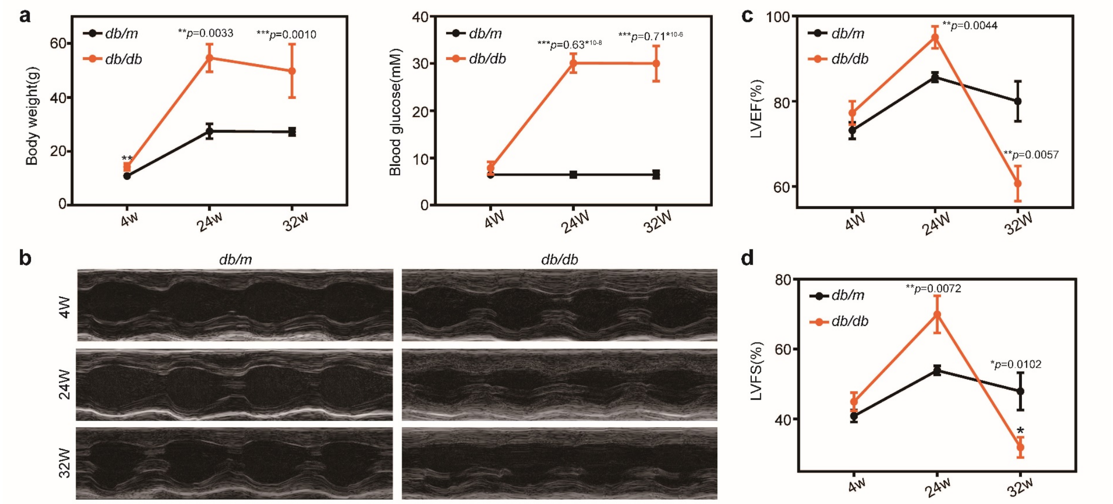

Supplementary Figure 1. Verification of the pathological status of type 2 diabetes mellitus (T2DM) mice at different ages (n means biologically independent animal). a. Body weight and blood glucose of *db/db* mice, n=5, * *vs db/m*. b. Echocardiography of *db/db* mice. c. left ventricular ejection fraction (LVEF) of *db/db* mice, n=3, * *vs db/m*. d. LVFS left ventricular fractional shortening (LVFS) of *db/db* mice, n=3, * *vs db/m*. Data are expressed as means ± SD. Two-tailed unpaired t-test was used for the analysis of statistical significance. Source data are provided as a Source Data file.

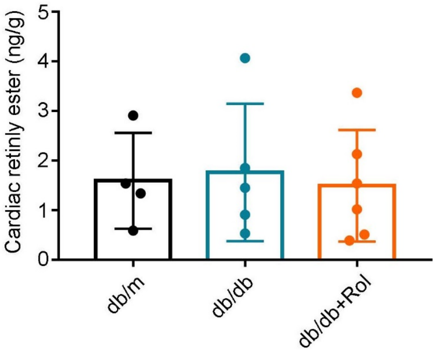

Supplementary Figure 2. Cardiac retinyl ester levels in T2DM mice supplemented with retinol (ROL) (n means biologically independent samples). Data are expressed as means ± SD. One-way ANOVA with Tukey post hoc test was used for the analysis of statistical significance. Source data are provided as a Source Data file.

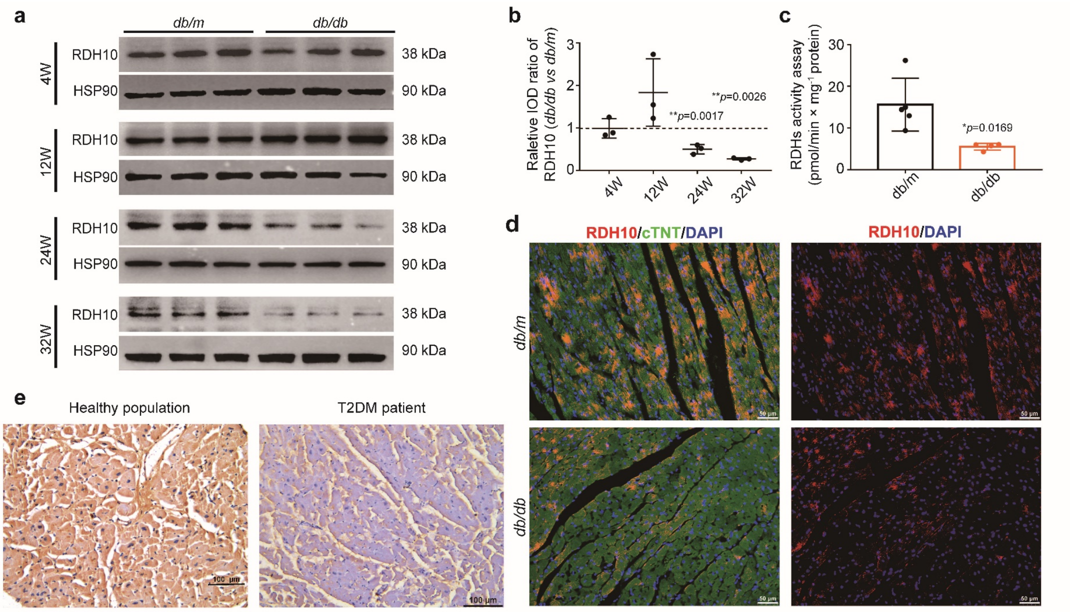

Supplementary Figure 3. Cardiac Retinol Dehydrogenase 10 (RDH10) expression in T2DM mice and patients (n means biologically independent samples). a. Western blotting (WB) images of cardiac RDH10 in *db/db* mice. b. Analysis of a, * *vs db/m*. c. Analysis of Retinol Dehydrogenases (RDHs) activity in *db/db* mice, * *vs db/m*. d. Immunofluorescence (IF) staining images of RDH10 in the heart of 32-week-old *db/db* mice, these results were independently repeated 3 times with similar results. e. Immunohistochemistry (IHC) staining images of RDH10 in the hearts of T2DM patients, these results were independently repeated 3 times with similar results. Data are expressed as means ± SD. Two-tailed unpaired t-test was used for the analysis of statistical significance. Source data are provided as a Source Data file.

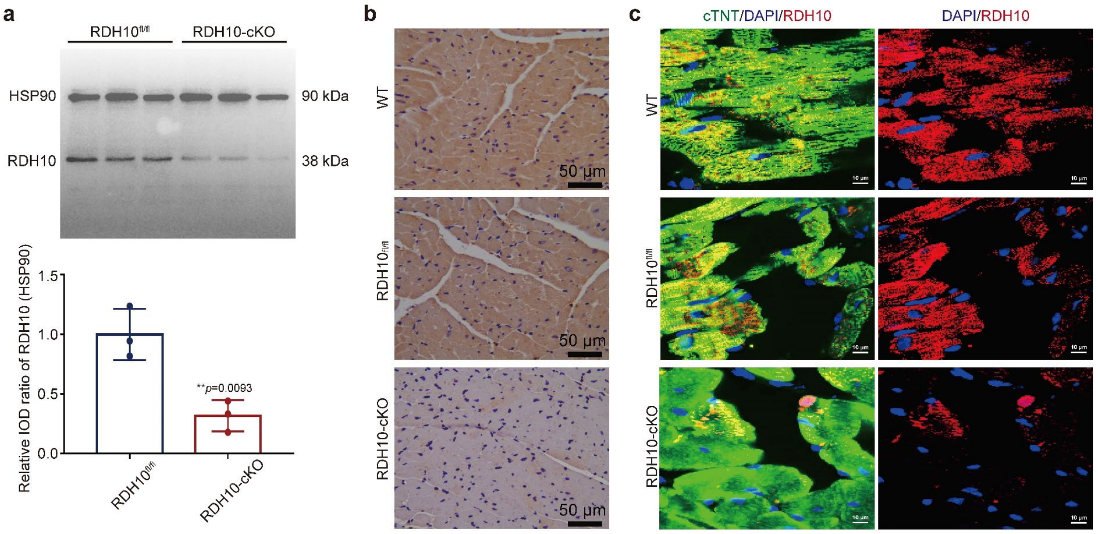

Supplementary Figure 4. Identification of RDH10-cKO mice (n means biologically independent samples). a. WB image of cardiac RDH10 in the hearts of RDH10-cKO mice, * *vs* RDH10$^{fl/fl}$. b. IHC staining image of RDH10 in the hearts of RDH10-cKO mice, these results were independently repeated 3 times with similar results. c. IF image of RDH10 in the hearts of RDH10-cKO mice, these results were independently repeated 3 times with similar results. Data are expressed as means ± SD. Two-tailed unpaired t-test was used for the analysis of statistical significance. Source data are provided as a Source Data file.

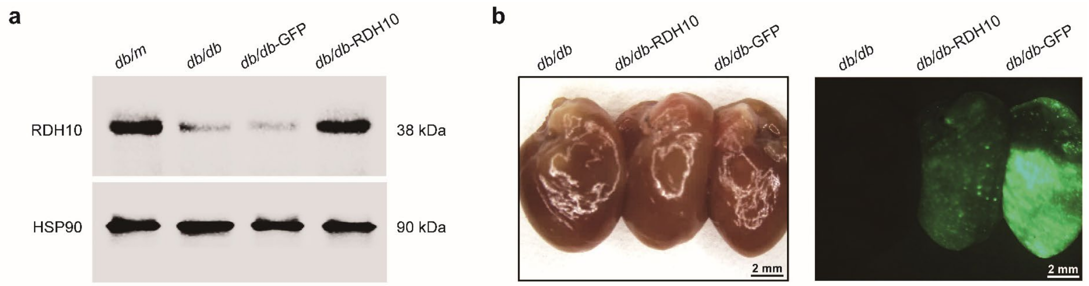

Supplementary Figure 5. Effect identification of RDH10 overexpression in the hearts of T2DM mice with adeno-associated virus 9 (AAV9)-RDH10 injection. a. Western blot images of cardiac RDH10 in *db/db* mice with AAV9, these results were independently repeated 3 times with similar results. b. Fluorescence detection in the hearts of *db/db* mice with AAV9, these results were independently repeated 3 times with similar results. Source data are provided as a Source Data file.

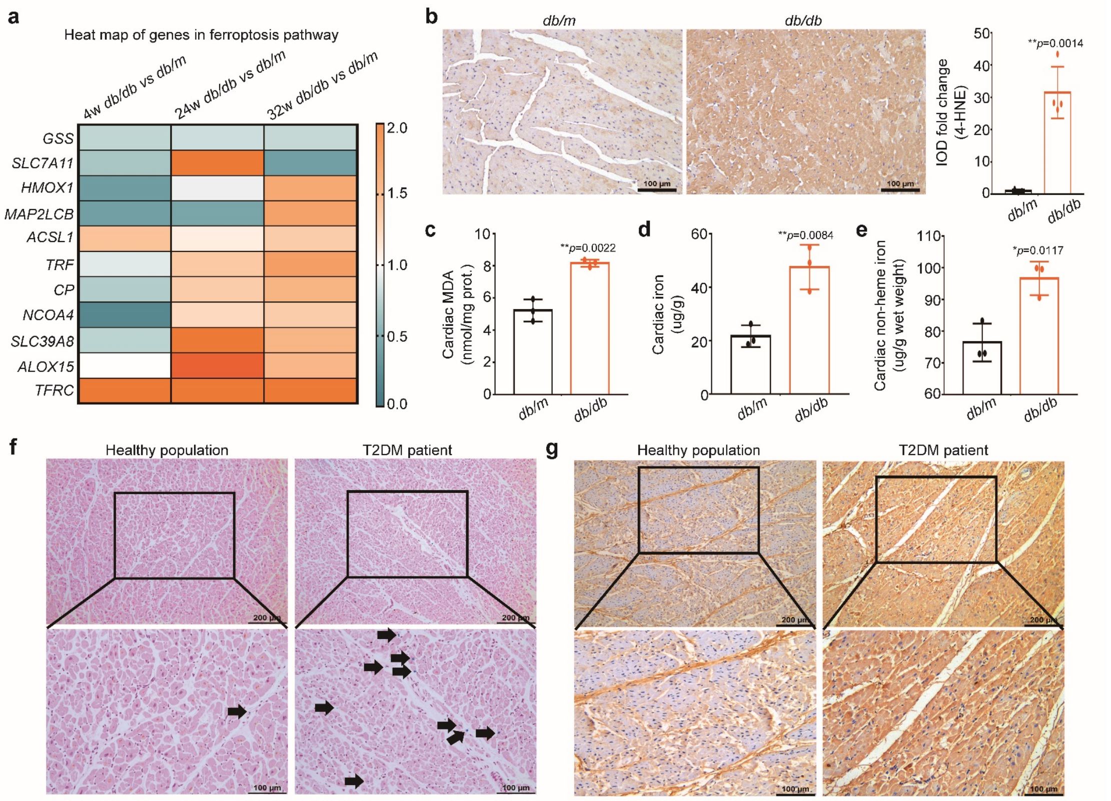

Supplementary Figure 6. Ferroptosis in the hearts of mice and patients with T2DM (n means biologically independent samples). a. Heat map of genes in ferroptosis pathway. b. Cardiac 4-Hydroxynonenal (4-HNE) staining, * *vs db/m*. c. Cardiac Malondialdehyde (MDA) levels, * *vs db/m*. d. Cardiac iron levels, * *vs db/m*. e. Cardiac non-heme iron levels, * *vs db/m*. f. Cardiac Perl's Prussian blue staining of T2DM patients, these results were independently repeated 3 times with similar results. g. Cardiac 4-HNE staining of T2DM patients, these results were independently repeated 3 times with similar results. (Black arrow: representative positive areas of Perl's Prussian blue staining) Data are expressed as means ± SD. Two-tailed unpaired t-test was used for the analysis of statistical significance. Source data are provided as a Source Data file.

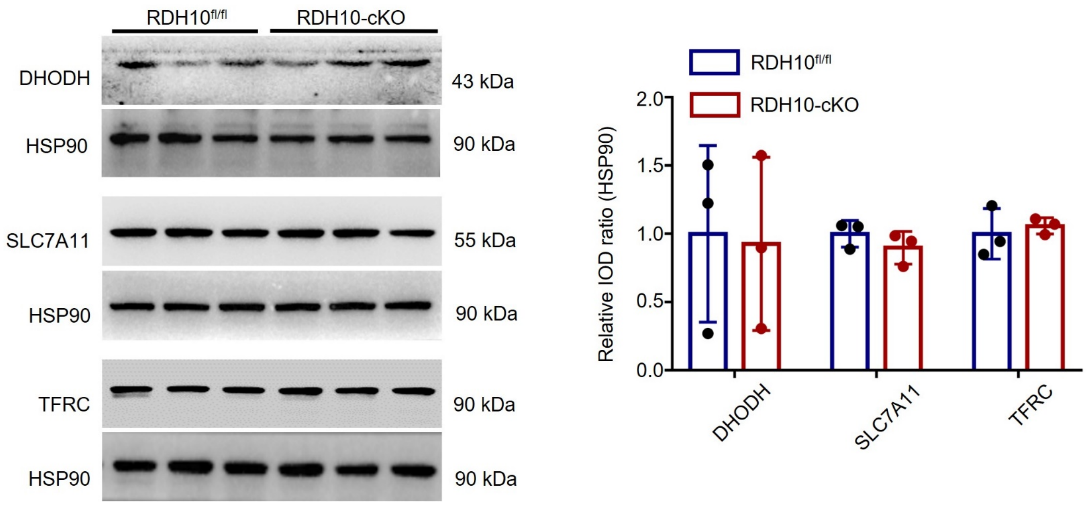

Supplementary Figure 7. Levels of other ferroptosis-related proteins in the heart of RDH10-cKO mice (n means biologically independent samples). Data are expressed as means ± SD. Two-tailed unpaired t-test was used for the analysis of statistical significance. Source data are provided as a Source Data file.

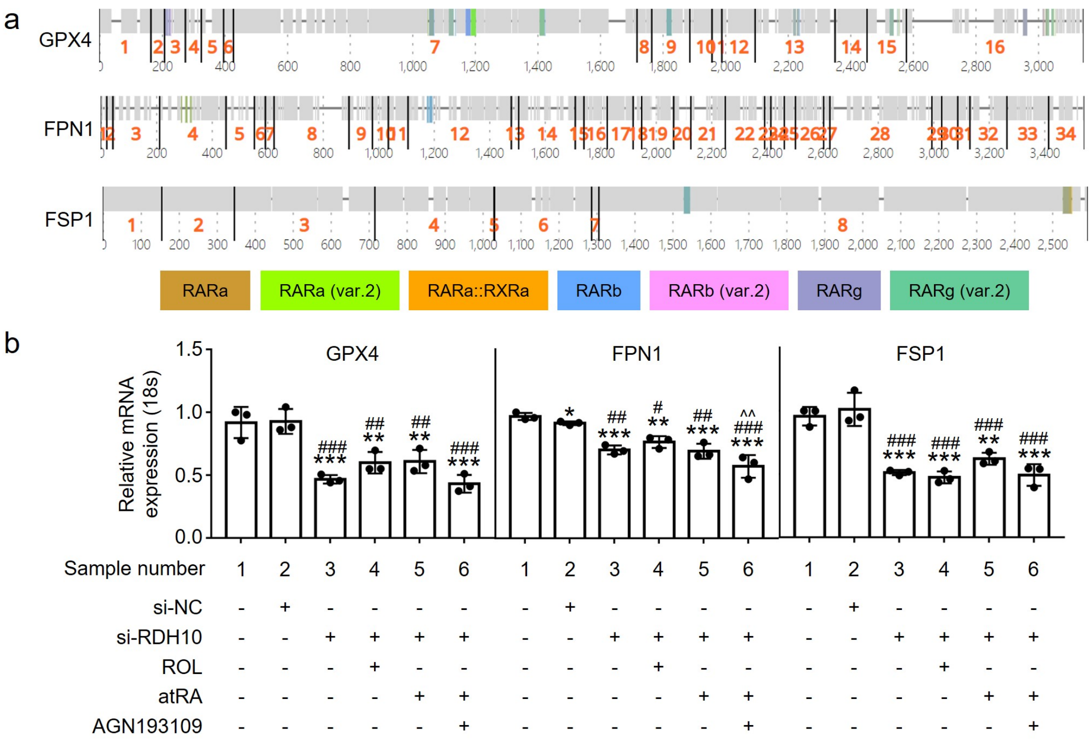

Supplementary Figure 8. Transcriptional regulation of GPX4, FPN1, and FSP1 by RARs (n means independent experiments). a. Binding site prediction of retinoic acid receptors (RARs) on promoters of GPX4, FPN1, and FSP1 (predicted by ConTra v3 [1]). b. mRNA levels of GPX4, FPN1 and FSP1 in NMPC, **p=0.0029 (GPX4, 4), 0.0036 (GPX4, 6), 0.004 (FPN1, 4) and 0.0017 (FSP1, 5), ***p=0.0002 (GPX4, 3), 0.0001 (GPX4, 6), 0.0005 (FPN1, 3), 0.0003 (FPN1, 5), 0.000008 (FPN1, 6), 0.0001 (FSP1, 3), 0.0001(FSP1, 4) and 0.0001 (FSP1, 6), *vs* 1; #p=0.0361 (FPN1, 4), ##p=0.0037(GPX4, 4), 0.0046(GPX4, 5), 0.0032 (FPN1, 3), 0.0022 (FPN1, 5), ###p=0.0002 (GPX4, 3), 0.0001 (GPX4, 6), 0.00004 (FPN1, 6), 0.00004 (FSP1, 3), 0.00002 (FSP1, 4), 0.0004 (FSP1, 5), 0.00003 (FSP1, 6), *vs* 2; ^^p=0.0068 (FPN1, 6), *vs* 4. Data are expressed as means ± SD. One-way ANOVA with Tukey post hoc test was used for the analysis of statistical significance. Source data are provided as a Source Data file.

Supplementary Table 1. The information of antibodies.

Antibodies	Manufacture and item NO.	Application (dilution rate)
RARa	CST (62294T)	WB (1:1000)
RARb	Abcam (ab53161)	WB (1:1000)
RARg	CST (8965T)	WB (1:1000)
RARa	Proteintech (10331-1-AP)	IHC (1:100)
RARb	Abcam (ab124701)	IHC (1:100)
RARg	Abcam (ab187159)	IHC (1:100)
HSP90	Proteintech (60318-1-Ig)	WB (1:1000)
RDH10	Proteintech (14644-1-AP)	WB (1:1000), IF (1:30), IHC (1:100)
CD36	Sigma (HPA002018)	WB (1:1000)
4-HNE	Abcam (ab46545)	IHC (1:200)
GPX4	Proteintech (14432-1-AP)	WB (1:500)
FPN1	Novus (NBP1-21502)	WB (1:1000)
SLC7A11	Proteintech (26864-1-AP)	WB (1:1000)
FSP1	Proteintech (20886-1-AP)	WB (1:1000)
DHODH	Proteintech (14877-1-AP)	WB (1:1000)
TFRC	Abcam (ab214039)	WB (1:1000)
cTNT	Invitrogen (MA512960)	IF (1:500)
Anti-Rabbit IgG(H+L)	Proteintech (SA00001-2)	WB (1:2500)
Anti-Mouse IgG(H+L)	Proteintech (SA00001-1)	WB (1:2500)
Anti-Mouse IgG (H+L) 488	Invitrogen (A-21202)	IF 1:2000
Anti-Rabbit IgG (H+L) 594	Invitrogen (A-21207)	IF 1:2000
HRP-labeled Anti-Rabbit IgG(H+L)	Beyotime (A0208)	IHC (1:100)
HRP-labeled Anti-Mouse IgG(H+L)	Beyotime (A0216)	IHC (1:100)

Supplementary Table 2. RT-PCR primers.

Gene	Forward primer	Reverse primer
GPX4	ATAAGAACGGCTGCGTGGTGAAG	TAGAGATAGCACGGCAGGTCCTTC
FPN1	TTGGTGACTGGGTGGATAAGAATGC	CGCAGAGGATGACGGACACATTC
FSP1	GACCCTTCCCTGGCAAGTTTAACG	CCTCCCACCACCACGATGAATTG
18S	CTCAACACGGGAAACCTCAC	CGCTCCACCAACTAAGAACG

Supplementary Table 3. Human sample donors' information.

	Healthy	T2DM	P value
Sex (Male/Female)	9/2	9/2	
Age (Years)	39.54±14.25	40.45±15.31	0.887
Visceral fat thickness (cm)	1.33±0.62	2.97±1.15	0.001
Heart weight (g)	328.18±67.20	385.45±71.33	0.067
Blood glucose control (Hypoglycemic drugs)	0/11	5/11	
Heart function (Normal)	11/11	1/11(4/11 N.A.)	

N.A.: No Answer.

Supplementary References

Kreft L, Soete A, Hulpiau P, Botzki A, Saeys Y, De Bleser P. ConTra v3: a tool to identify transcription factor binding sites across species, update 2017. *Nucleic acids research* **45**, W490-w494 (2017).
