# Supplementary material for: Retinol dehydrogenase 10 reduction mediated retinol metabolism disorder promotes diabetic cardiomyopathy in male mice
Source: Nat Commun. 2023 Mar 2;14:1181. doi: 10.1038/s41467-023-36837-x (PMC9981688; doi:10.1038/s41467-023-36837-x)
Supplement: Supplementary file 3 — Reporting Summary [file 41467_2023_36837_MOESM3_ESM.pdf]

nature portfolio

Corresponding author(s): Weibin Cai

Last updated by author(s): Jan 15, 2023

Reporting Summary

Nature Portfolio wishes to improve the reproducibility of the work that we publish. This form provides structure for consistency and transparency in reporting. For further information on Nature Portfolio policies, see our Editorial Policies and the Editorial Policy Checklist.

Statistics

For all statistical analyses, confirm that the following items are present in the figure legend, table legend, main text, or Methods section.

n/a	Confirmed	
☐	☒	The exact sample size (*n*) for each experimental group/condition, given as a discrete number and unit of measurement
☐	☒	A statement on whether measurements were taken from distinct samples or whether the same sample was measured repeatedly
☐	☒	The statistical test(s) used AND whether they are one- or two-sided<br>*Only common tests should be described solely by name; describe more complex techniques in the Methods section.*
☒	☐	A description of all covariates tested
☐	☒	A description of any assumptions or corrections, such as tests of normality and adjustment for multiple comparisons
☐	☒	A full description of the statistical parameters including central tendency (e.g. means) or other basic estimates (e.g. regression coefficient) AND variation (e.g. standard deviation) or associated estimates of uncertainty (e.g. confidence intervals)
☐	☒	For null hypothesis testing, the test statistic (e.g. *F*, *t*, *r*) with confidence intervals, effect sizes, degrees of freedom and *P* value noted<br>*Give P values as exact values whenever suitable.*
☒	☐	For Bayesian analysis, information on the choice of priors and Markov chain Monte Carlo settings
☒	☐	For hierarchical and complex designs, identification of the appropriate level for tests and full reporting of outcomes
☒	☐	Estimates of effect sizes (e.g. Cohen's *d*, Pearson's *r*), indicating how they were calculated

Our web collection on statistics for biologists contains articles on many of the points above.

Software and code

Policy information about availability of computer code

Data collection	Electrophoretic techniques were performed in a PowerPac/Mini (BIO-RAD, California, USA), and exposure of immunoblotting was performed in a BIO-RAD imaging system (BIO-RAD, California, USA). Q-PCR was performed on the ABI Q6 Flex Real-Time PCR machine (Applied Biosystems, Foster City, CA, USA). FFAs uptake was measured by a small animal living fluorescence imaging system (IVIS Spectrum, PerkinElmer, USA). Immunohistochemistry of tissues was examined and photographed with a microscope (DFC700T, Leica, Germany). Immunofluorescence images were obtained using a confocal microscopy (LSM 800, Zeiss), Transmission electron microscopy (TEM) was examined and photographed with a transmission electron microscope (Tecnai G2 Spirit Twin +GATAN 832.10W; FEI; Czech Republic), a Sciex Triple Quad TM 4500MD MS (Sciex, M.A., U.S.A), a Phenomenex Kinetex C18 (50*2.1mm, 2.6μm, Phenomenex, CA, USA), a Phenomenex Kinetex C18 (100*2.1mm, 1.7μm, Phenomenex, CA, USA) and a Spherisorb S3W column (4.6 mm × 100 mm; Waters). For RNA-seq, RNA and the library preparation integrity were inspected with Agilent Bioanalyzer 2100 (Agilent Technologies, Santa Clara, CA, USA), cluster and first dimension sequencing primer hybridization were accomplished on the cBot of the Illumina sequencing machine, sequencing performed at SHBIO Corporation. Echocardiography of mice were performed by a Vevo 3100 Imaging System (VisualSonics, Canada).
Data analysis	Quantifications of immunoblotting, immunofluorescence staining, and immunohistochemistry staining were analyzed by Image J (version 1.45) (National Institutes of Health, Bethesda, MD, USA) and Image-Pro Plus version 6.0 software （Media Cybernetics, Inc. , Rockville, MD, USA). Statistical analysis was performed with the GraphPad Prism 7 (Inc., La Jolla, CA) and R-4.2.2. LC-MS data were acquired and analyzed with Analyst® MD version 1.6.3 and Multi Quant TM MD version 3.0.2 (Sciex, M.A., U.S.A). The KOBAS (http://kobas.cbi.pku.edu.cn/) was used for KEGG pathway and GO terms analysis. The Biorender.com was used for produce Fig 9. The ConTra v3 (http://bioit2.irc.ugent.be/contra/v3/#/step/1) was used for RARs binding sites prediction in supplementary figure 8. Adobe Illustrator software (California, USA) was used for figures layout.

For manuscripts utilizing custom algorithms or software that are central to the research but not yet described in published literature, software must be made available to editors and reviewers. We strongly encourage code deposition in a community repository (e.g. GitHub). See the Nature Portfolio guidelines for submitting code & software for further information.

Data

Policy information about availability of data

All manuscripts must include a data availability statement. This statement should provide the following information, where applicable:

- Accession codes, unique identifiers, or web links for publicly available datasets
- A description of any restrictions on data availability
- For clinical datasets or third party data, please ensure that the statement adheres to our policy

Source data contained the raw data underlying the following types of display items: Each value corresponding to each statistical chart and uncropped versions of any gels or blots, labelled with the relevant panel and identifying information. The affymetrix raw files generated in this study have been deposited in the GEO database under accession code GSE202418 [https://www.ncbi.nlm.nih.gov/geo/query/acc.cgi?acc=GSE202418]. Source data are provided with this paper. The KOBAS (http://kobas.cbi.pku.edu.cn/) was used for KEGG pathway and GO terms analysis. The Biorender.com was used for produce Fig 9. The ConTra v3 (http://bioit2.irc.ugent.be/contra/v3/#/step/1) was used for RARs binding sites prediction in supplementary figure 8.

Human research participants

Policy information about studies involving human research participants and Sex and Gender in Research.

Reporting on sex and gender	In this study, we used human heart samples from 18 age-matched male (biological attribute) and 4 age-matched female (biological attribute), and our findings apply to all sex. We did not analyze the data we collected for sex differences because human heart samples are very difficult to collect and we could not obtain enough female samples.
Population characteristics	We compare patient with an age difference of up to 5 years with healthy individual as a group.
Recruitment	We use human heart samples collected from the National Center for Medico-legal Expertise of Sun Yat-sen University instead of recruiting any alive population in this study. We were unable to obtain information on whether each donor had taken vitamins, their medication history, etc.
Ethics oversight	The use of human heart samples was approved by the ethics committee of Zhongshan School of Medicine, Sun Yat-sen University with the protocol number (2019-B027) and all data and sample collection were in strict accordance with ethics guidelines of Zhongshan School of Medicine, Sun Yat-sen University. Informed consent was obtained from the legal representatives of the victims. The principles outlined in the Declaration of Helsinki were followed.

Note that full information on the approval of the study protocol must also be provided in the manuscript.

Field-specific reporting

Please select the one below that is the best fit for your research. If you are not sure, read the appropriate sections before making your selection.

☒ Life sciences ☐ Behavioural & social sciences ☐ Ecological, evolutionary & environmental sciences

For a reference copy of the document with all sections, see nature.com/documents/nr-reporting-summary-flat.pdf

Life sciences study design

All studies must disclose on these points even when the disclosure is negative.

Sample size	we based on pre-test to select sample sizes, which leading to determine statistically significant effects.
Data exclusions	No data were excluded.
Replication	Most expriments were replicated at least two times. All replications confirmed the findings.
Randomization	All mice enrolled in our study were of the same week of age and of similar body weight, and then randomly assigned to specific treatment groups.
Blinding	immunofluorescence staining and immunohistochemistry staining and quantification was double-blinded in collect images and quantification using ImageJ software or Image-Pro Plus version 6.0 software. Other data analysis was performed in a simultaneous and unbiased manner for all collected samples, where no blinding was necessary.

Reporting for specific materials, systems and methods

We require information from authors about some types of materials, experimental systems and methods used in many studies. Here, indicate whether each material, system or method listed is relevant to your study. If you are not sure if a list item applies to your research, read the appropriate section before selecting a response.

Materials & experimental systems

n/a	Involved in the study	
☐	☒	Antibodies
☒	☐	Eukaryotic cell lines
☒	☐	Palaeontology and archaeology
☐	☒	Animals and other organisms
☒	☐	Clinical data
☒	☐	Dual use research of concern

Methods

n/a	Involved in the study	
☒	☐	ChIP-seq
☒	☐	Flow cytometry
☒	☐	MRI-based neuroimaging

Antibodies

Antibodies used

RARa (E6Z6K) Cell signaling technology (62294T) Western blotting 1:1000
RARb Abcam (ab53161) Western blotting 1:1000
RARγ1 (D3A4) Cell signaling technology (8965T) Western blotting 1:1000
RARa Proteintech (10331-1-AP) IHC 1:100
RARb Abcam [EPR2017] (ab124701) IHC 1:100
RARg Abcam [EPR2020(N)] (ab187159) IHC 1:100
HSP90 Proteintech (60318-1-Ig) Western blotting 1:1000
RDH10 Proteintech (14644-1-AP) Western blotting 1:1000, IF 1:30, IHC 1:100
CD36 Sigma (HPA002018) Western blotting 1:1000
4-HNE Abcam (ab46545) IHC 1:200
GPX4 Proteintech (14432-1-AP) Western blotting 1:500
FPN1 (Ferroportin/SLC40A1 Antibody) Novus (NBP1-21502) Western blotting 1:1000
SLC7A11/xCT Proteintech (26864-1-AP) Western blotting 1:1000
AIFM2/ FSP1 Proteintech (20886-1-AP) Western blotting 1:1000
DHODH Proteintech (14877-1-AP) Western blotting 1:1000
TFRC Abcam (Transferrin Receptor antibody [EPR20584]) Western blotting 1:1000
cTNT (Cardiac Troponin T) Invitrogen (MA512960) IF 1:500
HRP-conjugated Affinipure Goat Anti-Rabbit IgG(H+L) Proteintech (SA00001-2) Western blotting 1:2500
HRP-conjugated Affinipure Goat Anti-Mouse IgG(H+L) Proteintech (SA00001-1) Western blotting 1:2500
Donkey anti-Mouse IgG (H+L) Highly Cross-Adsorbed Secondary Antibody, Alexa Fluor™ 488 Invitrogen (A-21202) IF 1:2000
Donkey anti-Rabbit IgG (H+L) Highly Cross-Adsorbed Secondary Antibody, Alexa Fluor™ 594 Invitrogen (A-21207) IF 1:2000
HRP-labeled Goat Anti-Rabbit IgG(H+L) Beyotime (A0208) IHC 1:100
HRP-labeled Goat Anti-Mouse IgG(H+L) Beyotime (A0216) IHC 1:100

Validation

Antibody validation information can be found on the following website:
RARa (E6Z6K) Cell signaling technology (62294T): https://www.cellsignal.com/products/primary-antibodies/rara-e6z6k-rabbit-mab/62294?site-search-type=Products&N=4294956287&Ntt=62294t&fromPage=plp&_requestid=2919279
RARb Abcam (ab53161): https://www.abcam.cn/retinoic-acid-receptor-beta-antibody-ab53161.html
RARγ1 (D3A4) Cell signaling technology (8965T): https://www.cellsignal.com/products/primary-antibodies/rarg1-d3a4-xp-rabbit-mab/8965?site-search-type=Products&N=4294956287&Ntt=8965t&fromPage=plp&_requestid=2919637
RARa Proteintech (10331-1-AP): https://www.ptglab.com/products/RARA-Antibody-10331-1-AP.htm
RARb Abcam [EPR2017] (ab124701): https://www.abcam.cn/retinoic-acid-receptor-beta-antibody-epr2017-ab124701.html
RARg Abcam [EPR2020(N)] (ab187159): https://www.abcam.cn/retinoic-acid-receptor-gamma-antibody-epr2020n-n-terminal-ab187159.html
HSP90 Proteintech (60318-1-Ig): https://www.ptglab.com/products/HSP90-Antibody-60318-1-Ig.htm
RDH10 Proteintech (14644-1-AP): https://www.ptglab.com/products/RDH10-Antibody-14644-1-AP.htm
CD36 Sigma (HPA002018): https://www.sigmaaldrich.com/HK/zh/product/sigma/hpa002018
4-HNE Abcam (ab46545): https://www.ncbi.nlm.nih.gov/pmc/articles/PMC4512214/
GPX4 Proteintech (14432-1-AP): https://www.ptglab.com/products/GPX4-Antibody-14432-1-AP.htm
FPN1 (Ferroportin/SLC40A1 Antibody) Novus (NBP1-21502): https://www.novusbio.com/products/ferroportin-slc40a1-antibody_nbp1-21502
SLC7A11/xCT Proteintech (26864-1-AP): https://www.ptglab.com/products/xCT-Antibody-26864-1-AP.htm
AIFM2/ FSP1 Proteintech (20886-1-AP): https://www.ptglab.com/products/AIFM2-Antibody-20886-1-AP.htm
DHODH Proteintech (14877-1-AP): https://www.ptglab.com/products/DHODH-Antibody-14877-1-AP.htm
TFRC Abcam (Transferrin Receptor antibody [EPR20584]): https://www.abcam.com/transferrin-receptor-antibody-epr20584-ab214039.html
cTNT Invitrogen (Cardiac Troponin T Monoclonal Antibody [13-11]): https://www.thermofisher.com/antibody/product/Cardiac-Troponin-T-Antibody-clone-13-11-Monoclonal/MA5-12960

Animals and other research organisms

Policy information about studies involving animals; ARRIVE guidelines recommended for reporting animal research, and Sex and Gender in Research

Laboratory animals	Db/mmice (C57BLKS/J background), db/db mice (C57BLKS/J background), and MYH6-iCre mice (C57BL/6 background) were purchased from GemPharmatech (Nanjing, Jiangsu, China). RDH10fl/fl mice (C57BL/6 background) were generously provided by Prof. Jianxing Ma (Health Sciences Center, University of Oklahoma). RDH10-cKO mice were generated by cross breeding of RDH10fl/fl mice and MYH6-iCre mice. All enrolled mice were male and aged 6-8 weeks. Mice maintained at the Center for Disease Model Animals of Sun Yat-sen University. Mice were housed on a 12 h light-dark cycle at 22–25 °C with 40–70% humidity and allowed free access to food and water except as noted.
Wild animals	The study did not involve wild animals.
Reporting on sex	In general, the reason for using male mice in animal experiments is to avoid interference with the female estrous cycle, but for our study, estrogen in female mice has an impact on studying diabetes. Therefore, only male mice were used in our animal studies.
Field-collected samples	The study did not involve field-collected animals.
Ethics oversight	All animal experiments were approved by the Animal Care and Ethics Committee of Zhongshan School of Medicine, Sun Yat-sen University, and followed the National Institutes of Health Guidelines on the Care and Use of Animals (the protocol number is SYSU-IACUC-2019-B027).

Note that full information on the approval of the study protocol must also be provided in the manuscript.
